# The *Escherichia coli* Outer Membrane β-Barrel Assembly Machinery (BAM) Anchors the Peptidoglycan Layer by Spanning It with All Subunits

**DOI:** 10.3390/ijms22041853

**Published:** 2021-02-12

**Authors:** Elisa Consoli, Jean-François Collet, Tanneke den Blaauwen

**Affiliations:** 1 Bacterial Cell Biology and Physiology, Swammerdam Institute for Life Science, University of Amsterdam, 1098 XH Amsterdam, The Netherlands; e.consoli@uva.nl; 2 de Duve Institute, Université Catholique de Louvain, B-1200 Brussels, Belgium; jean-francois.collet@uclouvain.be; 3 Walloon Excellence in Life Sciences and BIOtechnology (WELBIO), B-1200 Brussels, Belgium

**Keywords:** *Escherichia coli*, β-barrel assembly machinery, BAM complex, outer membrane, peptidoglycan, Braun’s lipoprotein, immunolabelling

## Abstract

Gram-negative bacteria possess a three-layered envelope composed of an inner membrane, surrounded by a peptidoglycan (PG) layer, enclosed by an outer membrane. The envelope ensures protection against diverse hostile milieus and offers an effective barrier against antibiotics. The layers are connected to each other through many protein interactions. Bacteria evolved sophisticated machineries that maintain the integrity and the functionality of each layer. The β-barrel assembly machinery (BAM), for example, is responsible for the insertion of the outer membrane integral proteins including the lipopolysaccharide transport machinery protein LptD. Labelling bacterial cells with BAM-specific fluorescent antibodies revealed the spatial arrangement between the machinery and the PG layer. The antibody detection of each BAM subunit required the enzymatic digestion of the PG layer. Enhancing the spacing between the outer membrane and PG does not abolish this prerequisite. This suggests that BAM locally sets the distance between OM and the PG layer. Our results shed new light on the local organization of the envelope.

## 1. Introduction

Diderm Gram-negative bacteria are characterized by the presence of two concentric membranes: the inner membrane (IM) that surrounds the cytoplasm, the outer membrane (OM) that encloses the IM, and the space in between, called the periplasm. The IM and the OM differ in lipid composition, in as much as the IM is a symmetric bilayer composed by phospholipids (PL), while the OM is asymmetrical with PL in the inner leaflet and lipopolysaccharides (LPS) exclusively in the outer leaflet. Both membranes are enriched with integral (intrinsic) and peripheral (extrinsic) proteins. The IM integral proteins have one or multiple membrane-spanning α-helices embedded in the PL bilayer, whereas the OM proteins (OMP) are channel-forming porins, consisting of transmembrane β-barrels. Membrane proteins are produced in the cytoplasm and transported to their envelope destination. IM protein insertion is facilitated by the proximity of the producers and transporters, that can use ATP-driven energy to pass them through the membrane. In contrast, ATP is not present in the periplasm. OMPs require transportation across the crowded aqueous periplasmic space with the help of soluble chaperones [1], that will deliver the substrates to their OM acceptor: the β-barrel assembly machinery (BAM) [2]. The BAM machinery recognizes the OMPs and inserts them in the OM. In *Escherichia coli*, BAM is composed of five subunits: a β-barrel structure, BamA, and four lipoproteins, BamB–E. BamA, the core of the complex, contains five polypeptide-transport associated (POTRA) domains in the periplasm. BamA and BamD are the only essential components and are well conserved amongst Gram-negative bacteria [2,3]. Single deletion of BamB, BamC, and BamE subunits cause different phenotypes: Δ*bamB* mutants have a generally strong OM defect [4], while Δ*bamC* and Δ*bamE* do not exhibit any severe abnormalities. Recently, it was found that BamE is important for the formation of complexes between RcsF, a stress envelope sensor, and BamA [5,6,7,8].

The BAM complex is the major component to ensure OM integrity, as all β-barrel proteins are dependent on it, including LptD, the essential β-barrel that inserts lipopolysaccharides in the outer leaflet of the OM. BAM is fundamental for protecting the bacteria from an enormous variety of hostile environments and from the presence of toxic compounds, such as antibiotics.

The OM encloses the biggest macromolecule of most bacteria: the sacculus, also known also as peptidoglycan (PG) or murein. The PG is a polymer of sugars and amino acids that forms a mesh-like structure. Its chemical composition and function are highly conserved amongst bacterial species. It provides shape, rigidity, and protection against internal turgor pressure [9]. Gram-negative bacteria have evolved sophisticated systems for maintaining the integrity and the functionality of the OM and the PG and coordinating their synthesis and turnover, in concert with cell elongation and cell division. During cell growth, the inhibition of PG production, using antibiotics, or its specific degradation, by lysozyme, result in cell lysis [10]. The physical tethering between OM and PG, covalently or not, is one of the strategies adopted to ensure coordination and communication between the two layers. In fact, PG is covalently crosslinked to the OM via the most abundant *E. coli* protein: Braun’s lipoprotein (Lpp) [11,12]. Lpp is a homotrimer of a 58 amino acid α-helical protein, with an N-terminal lipid tail embedded in the OM and the carboxy-terminus covalently connected to the peptidoglycan. The main role of Lpp consists of maintaining the physiological distance between the outer membrane and the murein, especially during cell division [13,14,15]. Despite the high production of Lpp, it is not an essential protein [16]. In 2017, it was shown that manipulating the length of Lpp increased the OM–PG distance artificially. Thus, a useful tool was created for testing relations between OM and PG itself [14,15].

Although BamA is embedded in the outer membrane, its POTRA domains extend into the periplasm, where they bind to the BAM accessory lipoproteins. How the POTRA domains and the BAM accessory lipoproteins are located with respect to the PG remains unknown. Here, by using immunofluorescence, we investigated the spatial organization of BAM within the periplasmic compartment. This information is essential for further understanding on how the OMPs pass through the PG to be inserted into the OM.

## 2. Results

### 2.1. The BAM Complex Is Evenly Distributed in the Envelope

**Figure 1:**
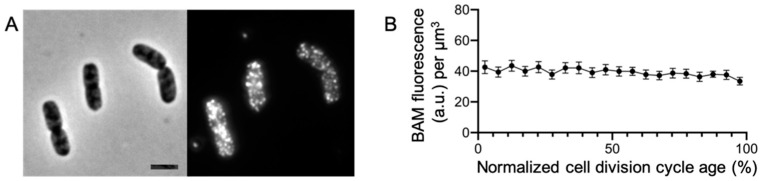
Barrel assembly machinery (BAM) immunofluorescence on *E. coli* wild-type cells. Phase-contrast and fluorescence microscopy images of anti-BAM (1:500) immunofluorescence on wild-type cells BW251113. The BAM complex localizes in bright distinct foci all along the cell periphery (**A**). The concentration of the complex, plotted against the cell age in %, indicates that the production of BAM is constant during the cell division cycle (**B**). The fluorescence concentration line connects 5% age bins with the 95% confidence range indicated by the error bars. The cells were grown in rich medium at 37 °C. Scale bar equals 2 μm. Number of analyzed cells was 1686.

To be able to investigate the BAM complex in relation to other cell compartments, we generated new polyclonal antibodies against the whole folded BAM machinery. The antibodies were made to be used for Western blot analysis and immunofluorescence localization studies. Their specificity was validated both with native and denatured complexes; upon denaturation, all subunits of the complex were recognized, albeit with different efficiencies (Figure S1). Then, the antibody affinity was tested for immunofluorescence, and anti-BAM (αBAM) was used to examine the envelope distribution of native BAM complex in fixed *E. coli* BW25113 wild-type cells (Figure 1A). As expected, the BAM complex localizes on the periphery of the cells in bright distinct foci [17,18]. The immunolabelled cells were analyzed with the ImageJ plugin ObjectJ [19] in order to observe whether the complex is subject to change in expression over time. The BAM concentration remains stable during the cell division cycle, suggesting a constant production of the complex (Figure 1B). Our immunolabelling revealed that the BAM complex is distributed all along the cell periphery in foci and it is continuously expressed to match a constant concentration in the envelope during the cell division cycle.

### 2.2. The Peptidoglycan Impairs BAM Immunolabelling

**Figure 2:**
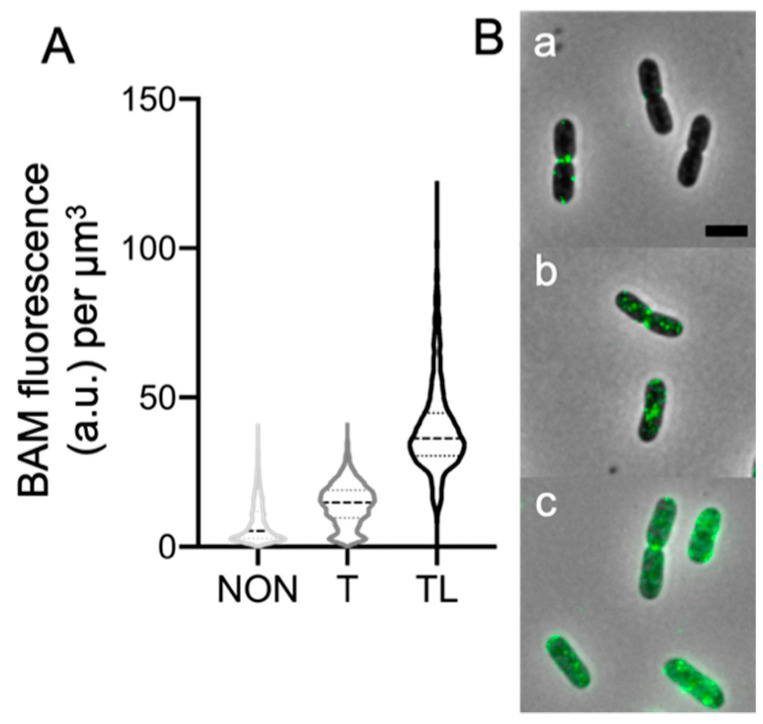
*E. coli* wild-type cells immunolabelled with αBAM using different permeabilization steps. The wild-type BW25113 cells were all immunolabelled against the BAM complex (1:500). The cells were unpermeabilized (NON, 1784 cells), permeabilized with Triton X-100 (T, 2021 cells), permeabilized with both Triton X-100 and lysozyme (TL, 1686 cells). The violin plots show that the different treatments exhibit diverse fluorescence intensities. The digestion of the peptidoglycan, in combination with the membrane permeabilization (TL), seems to be an essential treatment to reveal a significant amount of BAM complexes (**A**). In (**B**), images of cells unpermeabilized (**a**), permeabilized with Triton X-100 (**b**), permeabilized with both Triton X-100 and lysozyme (**c**). The cells were grown in rich medium at 37 °C. Scale bar equals 2 μm. This is a typical example of an experiment. The average percentage of αBAM fluorescence signal for Triton Χ-100 and Triton Χ-100 plus lysozyme treatment was 15 ± 7.4% and 85 ± 7.4%, respectively, for 6 independent experiments.

For the immunolabelling of Gram-negative cells, the OM is usually permeabilized with Triton X-100, to enable the entrance of the antibodies in the periplasmic space [20]. To ensure the detection of proteins located below the peptidoglycan layer, it is also needed to enzymatically digest the PG using lysozyme. In *E. coli*, the BamA subunit is embedded in the OM, facing both the external milieu and the periplasm, in which protrude the flexible 5 POTRA domains. The BAM accessory lipoproteins are all anchored in the OM, via a lipid tail on their amino-terminal cysteine. They extend in the periplasm, and, given their size, likely above the PG layer. To assess whether this is the actual situation, the wild-type strain BW25113 was permeabilized in different combinations. In order to reveal the epitopes accessible from the cell surface, those between the OM and PG, or below the PG layer, the cells were left untreated, treated with Triton X-100, or with both Triton X-100 and lysozyme, respectively, before the immunolabelling. The αBAM immunolabelling of the unpermeabilized bacteria barely showed any signal apart from a few bright spots, likely due to BamA and perhaps also to BamC, which has been reported to be surface-exposed (Figure 2 and Figure S2) [17]. Membrane permeabilization with Triton X-100 improved the detection of the BAM complex, compared to unpermeabilized cells, showing more foci along the cell axis and higher average fluorescence intensity, especially in higher concentration (Figure S3). However, the PG digestion with lysozyme seems to be an essential treatment to enable the exposure of a larger number of epitopes to the antibodies; epitopes that may be otherwise blocked by the peptidoglycan. These findings suggest that the BAM complex protrudes through or is in close proximity to the peptidoglycan layer.

### 2.3. Peptidoglycan Digestion Is Needed to Access Each BAM Subunit

**Figure 3:**
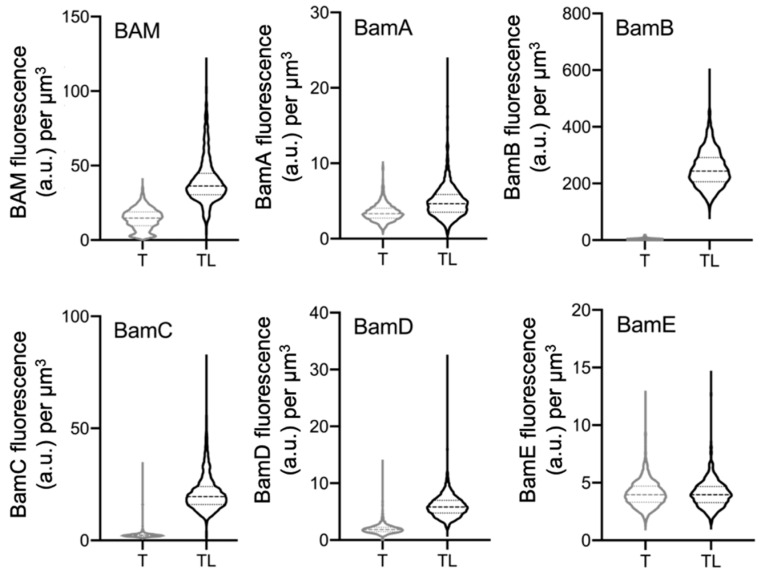
*E. coli* wild-type cells immunolabelled with antibodies against the single BAM subunits (αBamA–E). The wild-type BW25113 grown in rich medium, permeabilized with Triton X-100 only (grey) or in combination with lysozyme (black) and immunolabelled with αBAM and with antibodies specific for the single BAM subunits (1:500). Violin plots of the fluorescence concentration of the whole BAM complex and all the single BAM subunits (A–E). The different values underline that antibodies against the single BAM subunits exhibit a different affinity for their epitopes (A–D). Except for the αBamE (E) that shows poor binding, all the other subunits (A-D) give a higher signal only after the peptidoglycan digestion (TL). T is Triton X-100; TL is Triton X-100 and lysozyme. (BAM) *n* = 2021 T, *n* = 1686 TL; (A) *n* = 2021 T, *n* = 2042 TL; (B) *n* = 2036 T, *n* = 1450 TL; (C) *n* = 2014 T, *n* = 2373 TL; (D) *n* = 2157 T, *n* = 2029 TL; (E) *n* = 2029 T, *n* = 1654 TL.

The outer membrane BAM complex extends towards the cytoplasmic membrane and it is unknown whether it has contact with the PG layer. Calculating the size of BAM from its crystal structure [21,22] and relating this to the estimated distance between the OM and PG layer [23] suggests that it might be able to extend through the PG layer. The machinery is approximately 12 nm in length, 10 nm in width, and 14 nm in height. Subtracting the ±7 nm that is in the OM leaves 7 nm to reach the PG layer. The POTRA domains are arranged approximately on the same plane, aside from the POTRA 1 and 2 that protrude slightly towards the PG layer [22]. The thickness of the OM is around 6.87 ± 1.01 nm, the width of the PG is 6.35 ± 0.53 nm, and the space between OM and PG is 6.87 ± 1.01 nm [23]. Although these measurements predict BAM to be mostly above the PG layer, the large increase in immunofluorescence, after lysozyme treatment, suggests that the BAM machineries could reach and protrude through the PG layer. To determine whether one dominant epitope was causing this, wild-type *E. coli* cells were labelled using antibodies against the individual subunits. To increase antibodies’ specificity, those raised against BamB, BamC, and BamE were first pre-purified by adsorbing any non-specific IgG to their respective deletion strains. After the pre-purification, the supernatant, containing the remaining IgG molecules, was used to label BW25113 wild-type cells. Bacteria, then, were incubated with Triton X-100 only or in combination with lysozyme. Even though the single subunit antibodies do not always offer an optimal immunolabelling yield, from the fluorescence units’ concentration, it seems that the PG needs to be digested to access the BAM subunits (Figure 3 and Figure S4).

### 2.4. POTRA 1 Domain and BamE Require Peptidoglycan Digestion

**Figure 4:**
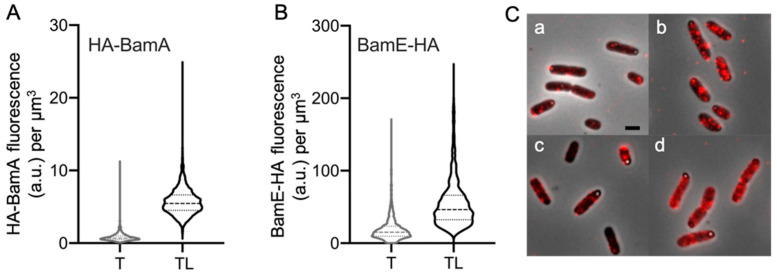
αHA tag immunolabelling. The proteins BamA and BamE, tagged with the HA epitope, were expressed in *E. coli* mutant cells *bamA101* and ΔbamE, respectively. The fixed cells were permeabilized with Triton X-100 only (T) or in combination with lysozyme (TL). The bacteria were then immunolabelled with αHA tag antibodies (1:100). The violin plots of the fluorescence concentration show that the lysozyme treatment is required to detect more HA-BamA (**A**) and BamE-HA (**B**) by the αHA tag antibodies. In **C**, fluorescent microscopy images of HA-BamA T (**a**), HA-BamA TL (**b**), BamE-HA T (**c**), and BamE-HA TL (**d**).The cells were grown in rich medium at 37 °C. HA-BamA: T *n* = 1786; TL *n* = 2431. BamE-HA: T *n* = 1494; TL *n* = 1082.

All BAM subunits seem to be shielded by the PG layer. Since we do not know the amino acid sequence of the epitopes recognized by the antibodies for the individual BAM subunits, a human influenza hemagglutinin (HA) tag was fused to the POTRA 1 domain of the BamA subunit or to the carboxy-terminus of the BAM lipoprotein BamE to obtain positional information. These two insertion points represent the furthest and the closest position to the OM, respectively. The proteins were expressed for four mass doublings from inducible low copy-number plasmids. After permeabilization with Triton X-100 only or in combination with lysozyme, the cells were immunolabelled with αHA tag specific antibodies (Figure 4). Sucrose gradient separation of the OM and the IM confirmed that BamE-HA is indeed in the OM (Figure S5). The analysis demonstrates that in both cases, the PG permeabilization is essential to make the HA tag accessible for the antibodies, suggesting that both HA tags are below or in the vicinity of the PG layer. The fusions assess whether the most murein-proximal POTRA domain and the smallest BAM subunit were both inaccessible by the presence of the murein layer.

### 2.5. Peptidoglycan Digestion Is Not Required for All OM-Lipoproteins

**Figure 5:**
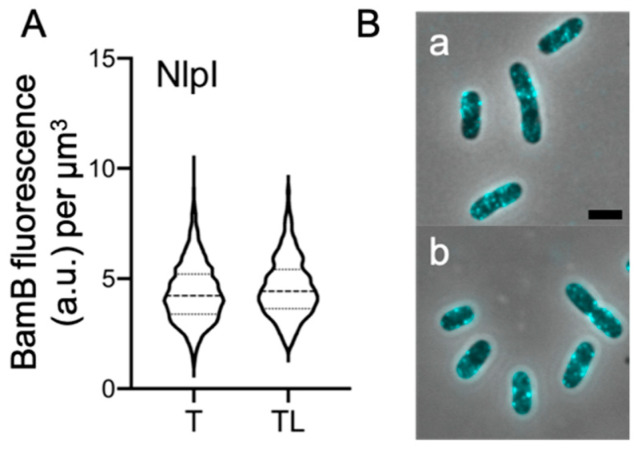
Immunolabelling of the outer membrane lipoprotein NlpI in *E. coli* wild-type cells. Wild-type BW25113 cells were permeabilized only with Triton X-100 (T, 1504 cells) or in combination with lysozyme (TL, 1015 cells). The cells were then immunolabelled with affinity-purified αNlpI antibodies (1:50). The violin plots of the fluorescence concentration show a negligible difference between the two NlpI signals (**A**). Fluorescent microscopy images of wild-type cells permeabilized with Triton X-100 only (**a**) and with Triton X-100 and lysozyme (**b**) immunolabelled with antibodies against NlpI (**B**). The NlpI concentration does not change between the two treatments. The cells were grown in rich medium at 37 °C. Scale bar equals 2 μm.

Our results strongly suggest that all the subunits of the BAM complex are immunolabelling-inaccessible due to the presence of the murein. We decided to test whether other OM lipoproteins give similar results. Therefore, the lipoprotein NlpI of wild-type *E. coli* cells was immunolabeled with specific antibodies [24]. NlpI is a dimeric lipoprotein that is distributed evenly over the cell envelope close to the OM [24]. No difference could be observed between the level of fluorescence or the distribution of foci in the cells treated with Triton X-100 only or treated with Triton X-100 and lysozyme (Figure 5). In fact, the detected NlpI concentration did not vary between the two samples (Figure 5A). This result indicates that the NlpI epitopes are accessible despite the presence of the murein layer, as lysozyme digestion is not required for detection. We can confidently conclude that not all of the lipoproteins’ immunolabelling is impaired by the presence of the PG. This also shows that the immunolabeling procedure does not press the PG layer against the OM.

### 2.6. Increasing the Distance between the Outer Membrane and Peptidoglycan Is Not Sufficient to Access the BAM Complex

**Figure 6:**
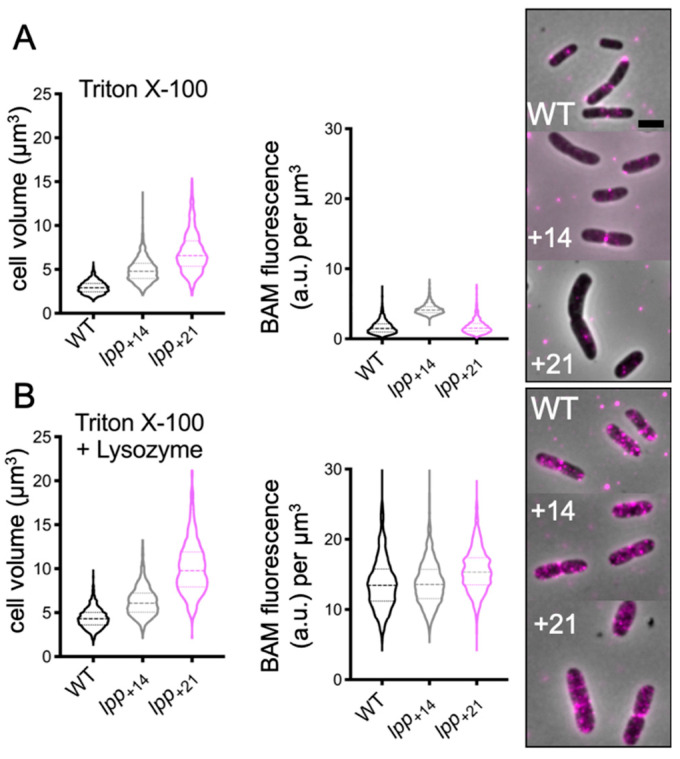
*E. coli* wild-type cells and its enlarged periplasmic derived mutants immunolabelled with αBAM. Violin plots of the BAM concentration of the wild-type MG1655 (WT, black), Lpp+14 (grey) and Lpp+21 (pink). After the peptidoglycan permeabilization (TL), the BAM signal increases about 3-fold amongst all the strains. The BAM signal is lower in cells with undigested peptidoglycan compared to the cells treated with lysozyme, irrespective of the enhanced distance between OM and PG. The cells were grown in rich medium at 37 °C. The antibodies were used in 1:500. A: wild-type *n* = 2062; Lpp+14 *n* = 1709; Lpp+21 *n* = 1991. B: wild-type *n* = 1697; Lpp+14 *n* = 1132; Lpp+21 *n* = 1141.

Our data indicate an intimate relationship between the BAM complex and the PG. The peptidoglycan layer has relatively large pores, which should allow the BAM subunits to pass through the layer [25,26]. We decided to push the peptidoglycan away from the OM, artificially increasing the spacing between the two layers. This should make the BAM epitopes accessible for the antibodies, despite the presence of the intact murein. *E. coli* strains were engineered to express a longer version of Braun’s lipoprotein (Lpp) with extensions of 14 and 21 amino acids. Lpp+14 and Lpp+21 strains were used to enhance the space between OM and PG with 3 and 5 nm, respectively [14]. The wild-type and its derivate variants Lpp+14 and Lpp+21 membranes were Triton X-100 permeabilized, in combination or not with lysozyme, then immunolabelled. The BAM complex concentration was quite similar in all three strains. Despite the greater distance between the OM and the PG in Lpp+14 and Lpp+21, the enzymatic digestion of the murein was still required to reveal a higher number of BAM complexes in the cells (Figure 6). The immunofluorescence results in Lpp+14 and Lpp+21 strongly suggest that an increase in OM–PG distance up to 5 nm does not change the spatial organization between the BAM and the PG layer. Based on our data, the murein seems to follow the BAM complex, despite the presence of elongated Lpp, suggesting that the OM–PG distance may vary along the cell axis.

## 3. Discussion and Conclusions

In this study, we tried to elucidate the spatial organization between the PG and the BAM complex. First, it was tested whether the BAM complex is located above the PG layer or in its close proximity, using different permeabilization steps for the immunolabelling assay. The results show clearly that BAM is not accessible to the antibodies, unless the murein is enzymatically digested first. The same outcome was obtained by immunolabelling the single subunits: the PG prevents the epitopes binding, resulting in a less efficient recognition by the specific antibodies. The BAM machinery is likely most of the time a permanent complex, as it can be easily isolated from the OM [22]; therefore, we assume that our immunolabeling reflects to a large extent the presence of the BAM complex and not of individual subunits.

The proximity between BAM and PG was further investigated by fusing BamE and the POTRA 1 domain of BamA with an HA-tag, which are the closest (±3 nm) and the furthest away (±7 nm) from the OM, respectively [22]. The recognition of the tagged BamA and BamE still needed the partial digestion of the PG layer in order to be bound by the αHA tag antibodies. This behavior seems specific for the BAM complex, since the 33 kDa OM-anchored lipoprotein NlpI does not need a partial digestion of the murein layer to be immunolabelled. To determine whether the BAM complex protrudes through the PG layer, the BAM localization was assayed in *E. coli* bacterial strains that express longer versions of Braun’s lipoprotein, enhancing the space between OM and the PG. Notwithstanding the increased distance (3–5 nm), the BAM complex was still not accessible without PG digestion. This result suggests that the PG layer has locally moved up towards the BAM complex, or that the OM with the BAM complex has moved down towards the PG to overrule the imposed distance by the Lpp. This would only be possible when the BAM complex is not able to move away from the PG layer, which suggests the presence of some kind of interaction. The BAM lipoproteins, for instance, could be positioned below the PG layer and together can anchor the murein, imposing a defined proximity between the OM and the PG.

The murein is elastic in the direction of the long axis, due to the orientation of the peptides [27]. Thus, the PG can assume different conformations that affect the diameter of its pores, also called tesserae [25,26]. In *E. coli*, the average radius of a tessera is about 2.06 nm, through which a globular uncharged protein of 25 kDa can easily pass, while in more stretched conditions or in two contiguous tesserae, a globular protein up to 50 kDa can diffuse through. All the BAM lipoproteins are in principle able to transverse the PG tesserae, in as much as the BamB subunit is approximately 40 kDa, the BamC subunit is 37 kDa, the BamD is 25 kDa, and the BamE is 12 kDa. BamB and BamD are bound directly to BamA. Nevertheless, BamD also interacts with BamC and BamE, forming a ~75 kDa subcomplex on their own. Assuming that all the subunits are located below the PG layer, these would be able to passively anchor the murein, imposing a defined distance between the OM and PG. The spatial organization between the BAM machinery and the murein should be advantageous for *E. coli*. For instance, the POTRA domain 1 interacts with the periplasmic chaperon SurA and is also able to bind unfolded OMPs, albeit non-specifically [28,29]. Having the POTRA domains positioned below the PG layer could help in achieving the proximity between chaperons and the BAM complex. The POTRA domains, in fact, are flexible [30,31,32] and each domain is 2 nm in width (PDB: 3EFC and 1M5Y), so they can freely diffuse through the PG. In support, recent studies revealed a super-complex, spanning from the IM to the OM, that involved the BAM machinery (BamA and BamB), the periplasmic chaperon SurA, the IM translocon (SecYEG), and its accessory interactors (PpiD, SecA or SecDF and YidC) [33,34]. Our findings could pave the way for better understanding how the OMPs can transverse the murein in order to be accommodated in the OM, a mechanism that is still unknown.

## 4. Materials and Methods

### 4.1. Bacterial Strains and Culture Conditions

The *Escherichia coli* K12 strains used here are listed in Table 1. The cells were cultured in rich medium (TY: 10g tryptone (Bacto Laboratories, Mount Pritchard NSW, Australia), 5 g yeast extract (Duchefa, Amsterdam, The Netherlands) and 5 g NaCl (Merck, Kenilworth, NJ, USA) per liter). Expression of HA-POTRA1-BamA and BamE-HA was induced with 15 μM isopropyl β-D-1-thiogalactopyranoside (IPTG, Promega, Fitchburg, WI, USA) from a pTrc99A-down promoter plasmid. Growth was measured by absorbance at 600 nm with a Biochrom Libra S70 spectrophotometer (Harvard Biosciences, Biosciences, Holliston, MA, USA). The bacterial strains were inoculated in 5 mL TY and grown overnight at 37 °C. Subsequently, the cultures were diluted 1:1000 in TY and kept at the OD_600_ value below 0.3. Fixation was performed with a final concentration of 2.8% formaldehyde and 0.04% glutaraldehyde for 15 min while shaking in the water bath at 37 °C, after which cells were harvested. The cells were then washed 3 times in phosphate buffered saline (PBS, 0.2 g KCI, 0.2 g KH_2_PO_4_, 8 g NaCI, 2.16 g Na_2_HPO_4_•7H_2_O per liter of distilled water, pH 7.3) to remove the excess fixative.

### 4.2. Antibodies Production

*E. coli* BL21[DE3] cells were transformed with plasmid carrying the subcomplex BamAB (pCDF-BamAB) and BamCDE (pET-BamCDE-His8) and grown in 500 mL lysogeny broth (LB) medium containing 100 μg·mL^−1^ ampicillin and 50 μg·mL^−1^ spectinomycin at 37 °C with shaking (200 rpm). Expression was induced with 0.4 mM IPTG when the culture reached an OD_600_ of 0.6 and growth was continued at 30 °C (150 rpm), then harvested after 2 h by centrifugation (7000× *g*, 15 min, 4 °C). Cells were resuspended in 14 mL of resuspension buffer (for 100 mL: 10 mM Tris-HCl pH 8.0, 3 mM EDTA, 1 tablet Protease Inhibitor Cocktail (Roche, Basel, Switzerland), DNaseI 10 μg·mL^−1^). The insoluble fraction was collected by centrifugation (5000× *g*, 30 min, 4 °C) and stored overnight at −80 °C. The day after, the pellet was resuspended in 14 mL of ice-cold resuspension buffer. The cells were disrupted by the high-pressure homogenizer Stansted for two rounds (1.7 mbar pressure). After eliminating the unbroken cells by centrifugation (5000× *g*, 15 min, 4 °C), the soluble fraction was ultracentrifuged (200,000× *g*, 30 min, 4 °C). The pellet was resuspended with 3 mL of DDM buffer (5 mM imidazole, 300 mM NaCl, 50 mM NaPO_4_, 1% DDM, and 10% glycerol) and dissolve by incubating overnight at 4 °C with gentle agitation, followed by centrifugation (90,000× *g*, 10 min, 4 °C). The supernatant was filtered on a 5 mL TALON Hi-TRAP column (GE Healthcare Life Science, Chicago, IL, USA), equilibrated with binding buffer (5 mM imidazole, 300 mM NaCl, 50 mM NaPO_4_, 0.05% DDM, and 10% glycerol). Peak fractions were concentrated to 500 μL using Vivaspin 2 (100 kDa MWCO) concentrators (GE Healthcare Life Science), snap-frozen in liquid nitrogen and stored at −80 °C. The purity of the BAM complex was analyzed on SDS-gel and the concentration was evaluated with a Bradford assay. The polyclonal rabbit antibodies were produced by Davids Biotechnologie GmbH (Regensburg, Germany), using 3 mg of the purified complex.

### 4.3. Immunolabelling

The membrane of the fixed cell population was permeabilized with 0.1% Triton X-100 in PBS (pH 7.2) at room temperature for 45 min. The murein was then digested by incubating the cells with PBS (pH 7.2) containing 100 μg·mL^−1^ lysozyme and 5 mM EDTA for 45 min at room temperature [20]. Non-specific binding sites were blocked by incubating the cells in 0.5% (*w*/*v*) blocking reagents (Boehringer, Mannheim, Germany) in PBS at 37 °C. Immunolabelling of the cells was performed with rabbit αBAM antibodies (1:500, Davids Biotechnologie GmbH) or αHA tag (1:100, Sigma-Aldrich, St. Louis, MO, USA) and were stained with Cy3 or AlexaFluor488 conjugated donkey αRabbit IgG (Jackson ImmunoResearch Laboratories, Inc., West Grove, PA, USA) diluted 1:300 in blocking reagent (Boehringer, Mannheim, Germany) according to the protocol [20].

### 4.4. Microscopy

For imaging, the cells were immobilized on 1% agarose in water slabs coated object glasses as described [39] and photographed with a Hamamatsu ORCA-Flash-4.0 (Hamamatsu, Naka-ku, Japan) CMSO camera mounted on an Olympus BX-60 Fluorescence microscope (Tokyo, Japan) though a UPlanApo 100x*/N.A*. 1.35 oil Iris Ph3 objective. Images were acquired using the Micromanager 1.4 plugin for ImageJ (version 1.4, https://www.micro-manager.org (accessed on 11 February 2021)) [40]. The fluorescence filter cubes used were: U-MNG (Cy3 ex560/40, dic585LP, em630/75) and EN-GFP (AlexaFluor488, ex470/40, dic495LP, em525/50).

### 4.5. Image Analysis

In all experiments, the cells were first imaged in phase contrast mode and then in fluorescence mode, and the images were then combined in hyper-stacks using ImageJ (version 1.53, http://imagej.nih.gov/ij/ (accessed on 11 February 2021)). Phase contrast and fluorescence images were juxtaposed with the fast Fourier transform as described by Turner et al. [41]. The fluorescence background was subtracted using the modal-values from the fluorescence images. Quantification of cellular localization patterns were obtained using the ObjectJ plugin of ImageJ (version 1.05, https://sils.fnwi.uva.nl/bcb/objectj/ (accessed on 11 February 2021)) [19]. The images were scaled to 15.28 pixels per μm.

### 4.6. Construction of HA Tagged BAM Subunits

The *E. coli* primers used in this study are listed in Table 2. The plasmid pEC-HA-BamA was constructed as follows. The *bamA* gene was first mutagenized in order to remove the internal *Nco*I restriction site, performing a quick site-direct PCR mutagenesis using primers 100-EC and 101-EC, obtaining the plasmid pEC-BamB-BamA*NcoI. The resulting product was treated with *Dpn*I to digest the methylated template plasmid. The HA tag sequence was inserted in between the native signal sequence of the *bamA* gene and the POTRA domain 1, amplifying the whole plasmid pEC-BamB-BamA*NcoI, using the primers 110-EC and 111-EC. Finally, the so-modified *bamA* gene was amplifying with the primers 106-EC and 107-EC, in order to insert at the extremities *Nco*I and *EcoR*I restriction sites. Next, the fragment was ligated into the vector pSAV057-OmpA177-mNG [42], to generate the plasmid pEC-HA-BamA.

pEC-BamE-HA was made by amplifying the *bamE* sequence with the primers 127-EC and 128-EC, to obtain a *Nco*I and an HA-*Hind*III site at the gene extremities, 5′ and 3′, respectively. The amplified sequence replaced the *ompA177-mNG* sequence by ligation into plasmid pSAV057-OmpA177-mNG [42]. All restriction enzymes used were purchased from New England Biolabs Inc. (Ipswich, MA, USA).

## Figures and Tables

**Table 1:** Strains and their genotypes used in this study.

Strain	Genotype	Reference 3
BW25113	*lacI*^+^*rrnB*_T14_ Δ*lacZ*_WJ16_ *hsdR*514Δ*ara-BAD*_AH33_ Δ*rhaBAD*_LD78_ *rph-1* Δ*(araB–D)567* Δ*(rhaD–B)568* Δ*lacZ4787*(::*rrnB-3*) *hsdR514 rph-1*	[16]
BL21DE3	F^–^ *omp*T *hsdS_B_* (r_B_–, m_B_–) *gal dcm* (DE3)	[35]
Δ*bamE*	BW25113 Δ*bamE::cam*	[36]
*bamA101*	DH300 *bamA101*	[37]
MG1655	F*^−^*, *lambda*^−^, *rph-1*	[38]
Lpp_+14_	DH300 Δ*lpp::lpp_+14_*	[14]
Lpp_+21_	DH300 Δ*lpp::lpp_+21_*	[14]

**Table 2:** Primers used in this study.

Name	**Primer Sequence**
100-EC- BamA*NcoI-FW	GCTGTAGGCGGTAACGCGATGGCGGTTGC
101-EC- BamA*NcoI-RV	GCTGTAGGCGGTAACGCGATGGCGGTTGC
106-EC-BamA-NcoI-FW	CGTATACCATGGCGATGAAAAAGTTGCTCATAGCGTCG
107-EC-BamA-EcoRI-RV	CGTAATGAATTCCCAGGTTTTACCGATGTTAAACTGGAAC
110-EC-HA-BamA-FW	GTGCCGGATGTGCCGGATTATGCGTTCGTAGTGAAAGATATTCATTTCGAAGGCCTTC
111-EC-HA-BamA-RV	CACATCCGGCACATCATACGGATACCCTTCAGCACCGTATACGGTGGC
127-EC-BamE-NcoI-FW	GCGCGCCATGGGCCGCTGTAAAACGCTGACTGC
128-EC-BamE-HA-HindIII	GCGCGAAGCTTTTAAGCGTAATCTGGAACATCGTATGGGTAGTTA CCACTCAGCGCAGGTTTGTTATC

## Data Availability

The data that support the findings of this study are available from the corresponding author upon reasonable request.

## Supplementary Materials

Supplementary materials can be found at https://www.mdpi.com/1422-0067/22/4/1853/s1.
