# Supplementary material for: The Escherichia coli Outer Membrane β-Barrel Assembly Machinery (BAM) Anchors the Peptidoglycan Layer by Spanning It with All Subunits
Source: Int J Mol Sci. 2021 Feb 12;22(4):1853. doi: 10.3390/ijms22041853 (PMC7918090; doi:10.3390/ijms22041853)
Supplement: Supplementary file 1 [file ijms-22-01853-s001.pdf]

International Journal of
Molecular Sciences

MDPI

Article

The *Escherichia coli* outer membrane β-Barrel Assembly Machinery (BAM) anchors the peptidoglycan layer by spanning it with all subunits.

Elisa Consoli [1], Jean-François Collet [2,3] and Tanneke den Blaauwen [1,*]

1 Bacterial Cell Biology and Physiology, Swammerdam Institute for Life Science, University of Amsterdam, NL; e.consoli@uva.nl
2 de Duve Institute, Université catholique de Louvain, Brussels, BE; jean-francois.collet@uclouvain.be
3 WELBIO, Brussels, BE.
* Correspondence: t.denblaauwen@uva.nl.

1. Supplementary Figures

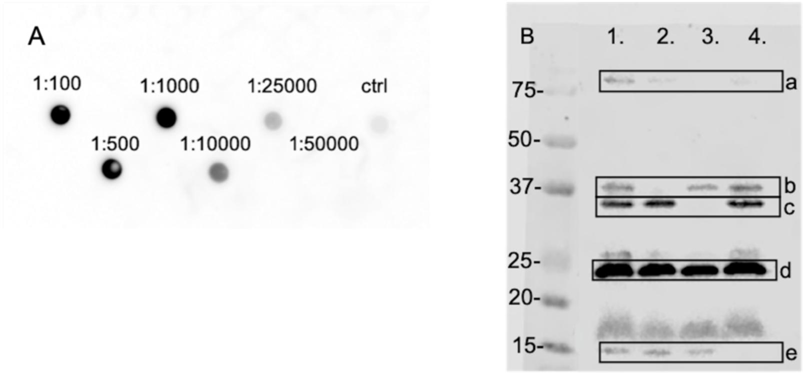

Figure S1. Screening of αBAM polyclonal antibodies. (**A**) Dot blot assay of the rabbit sera. The folded purified BAM complex has been blotted onto the membrane. Top and bottom rows: dilutions of serum from rabbit immunized with the purified BAM complex. Control: serum from the pre-immune animal. (**B**) Western blot analysis of the αBAM polyclonal antibodies specificity on the whole-cell extract. Parental wildtype strain BW25113 (1), Δ*bamB* (2), Δ*bamC* (3) and Δ*bamE* (4). The rectangles indicate the bands for BamA (a), BamB (b), BamC (c), BamD (d) and BamE (e). The specificity is confirmed by the knockout strains.

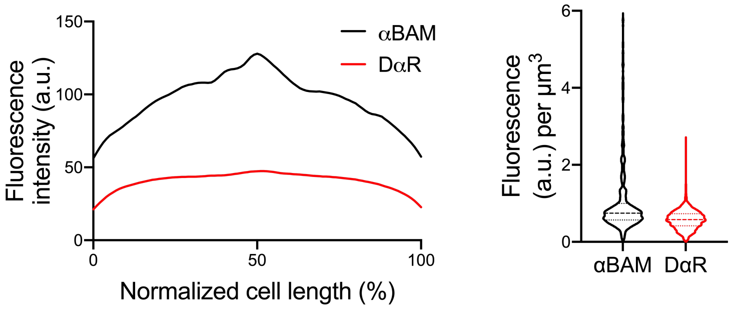

Figure S2. Unpermeabilized cells immunolabelled with αBAM and Donkey-αRabbit antibodies. The immunolabelling was performed on BW25113 wild-type cells grown in rich medium at 37 ºC. To prove that the αBAM recognizes some surface-exposed epitopes of BamA and BamC, the fixed cells were left unpermeabilized, then immunolabelled with the polyclonal αBAM and the secondary antibody Donkey-αRabbit (DαR), or only with DαR. The αBAM offers a different pattern of binding compared to the nonspecific binding of the secondary antibody alone, an indication of the recognition of some outer membrane surface-exposed accessible epitopes.

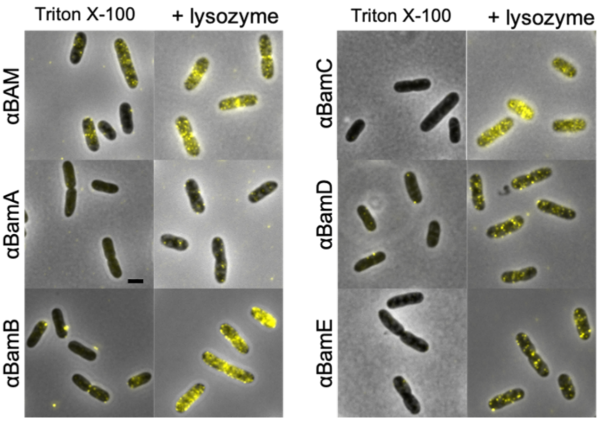

Figure S3. *E. coli* wild-type cells immunolabelled with antibodies against the single BAM subunits (αBamA-E). The wild-type BW25113 grown in rich medium, permeabilized with Triton X-100 only or in combination with lysozyme (left and right pannel of each immunolabeling, respectively) and immunolabelled with αBAM and with antibodies specific for the single BAM subunits (1:500). The microscopy images show a higher fluorescence after lysozyme treatment. The cells were grown in TY rich medium at 37 °C. Scale bar equals 2 µm.

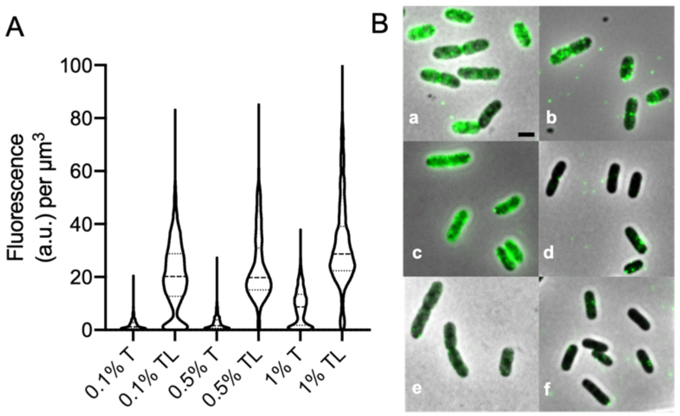

Figure S4. BAM immunolabelling on increasing outer membrane permeabilization. BW25113 *E. coli* wild type cells grown in rich medium, were permeabilized with Triton X-100 only (T) or in combination with Lysozyme (TL). The cells were permeabilized with different Triton X-100 concentrations: standard (0.1%), or 5-fold (0.5%) and 10-fold (1%). In **A**, the violin plots of the BAM fluorescent concentration suggest that the lysozyme treatment is essential to reveal a significant amount of BAM complexes. The cells incubated with 1% Triton X-100 show a higher BAM signal compared to the other concentrations, although not as much as the cells with digested peptidoglycan. In **B**, fluorescence microscopy images of cells permeabilized with 1% Triton X-100 and lysozyme (a), 1% Triton X-100 only (b), 0.5% Triton X-100 and lysozyme (c), 0.5%% Triton X-100 only (d), 0.1% Triton X-100 and lysozyme (e) and 0.1 % Triton X-100 only (f). The cells were grown in TY rich medium at 37 °C. Scale bar equals 2µm. 0.1% T n = 1601, 0.1% TL n = 2437, 0.5% T n = 1546, 0.5% TL n = 1161, 1% T n = 2283 and 1% TL n = 2094.

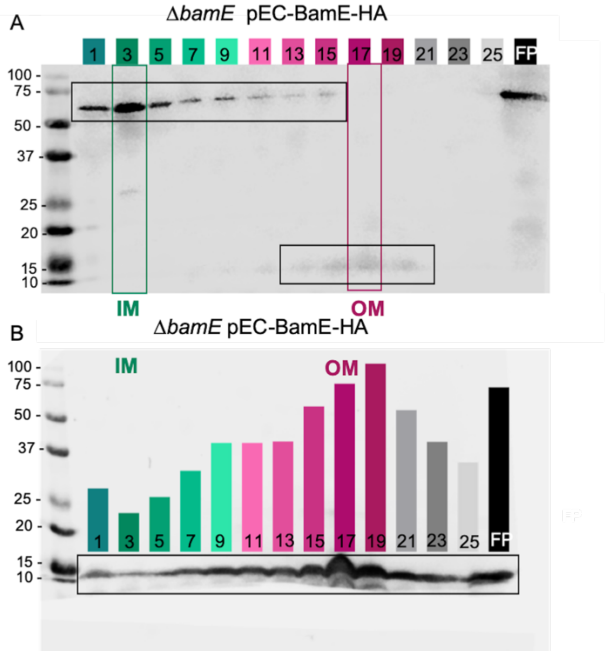

Figure S5. Localization of BamE-HA fusion. The western blot was performed on sucrose gradient centrifugation fractions of Δ*bamE* cells, grown in rich medium at 37 ºC, expressing the fusion BamE-HA (15 µM IPTG). Numbers 1-25 indicate the fractions taken plus the starting whole-cell extract (FP) for immunoblotting. (**A**) The IM marker αSecF (1:5000) and the OM indicator αLpp

(1:5000) were used to define the membrane fractions. The bars from left (green) to right (pink) indicate IM (1-9) and OM (11-19), respectively. (**B**) Western blot with αHA tag antibodies (1:5000). To help to visualize the migration shift, the blotting signal was used to quantify the enriched fractions of the tagged BamE subunit, as normalized bar charts. The OM lipoprotein BamE is not retained in the IM, therefore the immunolocalization is valid for the subcellular localization purpose.
